# Exploring Azithromycin’s Neuroprotective Role in Traumatic Brain Injury: Insights into Cognitive and Motor Recovery and Neuroinflammatory Modulation

**DOI:** 10.3390/ph18010115

**Published:** 2025-01-16

**Authors:** Mohannad A. Almikhlafi, Nehad A. Abdallah, Aakash Kumar, Tarun Sharma, Zuber Khan, Haifa A. Fadil, Sultan Althagfan, Ahmed K. B. Aljohani, Sara A. Almadani, Samar F. Miski, Tahani Saeedi, Rayan S. Alharbi, Abdulrahman M. Al-Harthe, Mohammed H. Alsubhi, Hanaa Wanas, Ahmed Aldhafiri, Sidharth Mehan, Hossein M. Elbadawy

**Affiliations:** 1 Department of Pharmacology and Toxicology, College of Pharmacy, Taibah University, Madinah 41477, Saudi Arabia; smadani@taibahu.edu.sa (S.A.A.); smiski@taibahu.edu.sa (S.F.M.); tahsaedi@taibahu.edu.sa (T.S.); alhray010@gmail.com (R.S.A.); dhmee.3007@gmail.com (A.M.A.-H.); mhsobhi@taibahu.edu.sa (M.H.A.); hanaa.wanas@kasralainy.edu.eg (H.W.); adhafiri@taibahu.edu.sa (A.A.); 2 Department of Pharmacognosy and Pharmaceutical Chemistry, College of Pharmacy, Taibah University, Madinah 41477, Saudi Arabia; nehad.amin@gmail.com (N.A.A.); akjohani@taibahu.edu.sa (A.K.B.A.); 3 Division of Neuroscience, Department of Pharmacology, ISF College of Pharmacy, IK Gujral Punjab Technical University, Jalandhar 144603, Punjab, India; kaakash526@gmail.com (A.K.); medtarun12@gmail.com (T.S.); zuber3491@gmail.com (Z.K.); sidharthmehan@isfcp.org (S.M.); 4 Department of Pharmacy Practice, College of Pharmacy, Taibah University, Madinah 41477, Saudi Arabia; hfadil@taibahu.edu.sa (H.A.F.); sthqfan@taibahu.edu.sa (S.A.); 5 Department of Medical Pharmacology, Faculty of Medicine, Cairo University, Cairo 11956, Egypt; 6 Health and Life Center, Taibah University, Madinah 41477, Saudi Arabia

**Keywords:** azithromycin, traumatic brain injury, neuroprotection

## Abstract

Background: Traumatic brain injury (TBI) is a leading cause of mortality worldwide and often results in substantial cognitive, motor, and psychological impairments, triggering oxidative stress, neuroinflammation, and neurodegeneration. This study examined the neuroprotective effects of azithromycin (AZI) in TBI. Methods: TBI was induced in rats using the weight-drop method. Subsequently, rats received a daily intraperitoneal (I.P.) dose of AZI (150 mg/kg) for 28 days. Behavioral tests (Morris water maze, rotarod, and open field tests) were performed to assess cognitive and motor functions. Neurochemical analyses included oxidative stress markers (GSH, SOD, MDA, catalase), inflammatory cytokines (TNF-α, IL-1β), apoptotic markers (caspase-3, Bax, Bcl-2), mitochondrial complexes (complex I, II, III, IV, and V), and the transforming growth factor- beta (TGF-β) as a neurofilament marker. Histological evaluations focused on neuronal integrity in the cortex, hippocampus, and striatum. Results: Treatment with AZI significantly facilitated motor and cognitive function recovery in TBI-affected rats. At the molecular level, AZI effectively reduced oxidative stress markers, ameliorated neuroinflammation by decreasing TNF-α, IL-1β, and neuronal apoptosis, and differentially modulated mitochondrial complexes. Histological assessments revealed enhanced neuronal integrity and fewer pathological changes in AZI-treated rats compared to untreated TBI controls. Conclusions: AZI was shown to interfere with several pathways involved in TBI’s pathophysiology. While preclinical results are promising, further studies are necessary to establish the long-term safety and efficacy of AZI in a clinical setting. This research supports the potential re-purposing of AZI as a novel treatment strategy for TBI and related neurodegenerative disorders.

## 1. Introduction

Traumatic brain injury (TBI) is a disruption of brain function caused by an external force, which can be either penetrating or non-penetrating. The symptoms can range from headaches, dizziness, confusion, and memory problems to brain death, coma, and eventually death [1]. About 2.8 million TBI-related emergency visits occur in the United States each year, making it a serious health concern that affects young children, adolescents, and older adults.

Falls are the primary cause of TBI, followed by being struck by objects or motor vehicle accidents [1]. TBI causes primary mechanical damage and secondary biochemical injury cascades, including inflammation, excitotoxicity, oxidative stress, and apoptosis [2,3]. Diffuse axonal injury in TBI causes extensive axonal damage that is hard to detect using conventional imaging techniques, but focal injuries in the orbitofrontal cortex and anterior temporal lobes lead to specific deficits in social behavior, emotion regulation, and decision-making [4]. TBI is associated with the accumulation of neurodegenerative proteins such as tau, β-amyloid, α-synuclein, and TAR DNA-binding 43 proteins (TDP-43), which significantly increase the risk of dementia, particularly Alzheimer’s disease, and chronic traumatic encephalopathy [5]. Additionally, the injured brain tissue triggers an immune response that leads to inflammation. At the same time, a complex interplay of neurotransmitters, biochemical mediators, and cytokines causes tissue damage through excitotoxicity, calcium dysregulation, nitric oxide production, and oxidative stress, ultimately resulting in apoptosis [6].

Antibiotics, usually part of the treatment of TBI patients, are used to prevent a post-injury infection that may follow an open head injury. A low mortality rate has been associated with the early administration of antibiotics in patients with TBI [7]. Among these antibiotics, azithromycin (AZI), a macrolide antibiotic, stands out not only for its antibacterial properties but also for its additional neuroprotective effects. In a spinal cord injury animal model, AZI exhibited anti-inflammatory and immunomodulatory actions. AZI reduces neuroinflammation and promotes the transition of macrophages to a protective M2 phenotype, which is crucial for tissue recovery following spinal cord injury [3]. AZI was found to be able to prevent axonal injury by averting the death of oligodendrocyte progenitor cells (OPCs) and improving their survival in hypoxic–ischemic brain injury [4]. Furthermore, AZI’s role in suppressing the production of pro-inflammatory cytokines, such as tumor necrosis factor alpha (TNF-α), interleukin 1 β (IL-1β), and interleukin 6 (IL-6), by inhibiting signal transducer and activator of transcription 1 (STAT1) and nuclear factor kappa-light-chain-enhancer of activated B cells (NF-κB) signaling pathways has been documented in a spinal cord injury rodent model [5]. Also, AZI has been seen to upregulate the expression of genes involved in mitochondrial oxidative phosphorylation (OXPHOS) enzymes, mitochondrial biogenesis factors, antioxidants, HIF1α, and glycolytic enzymes in human fibroblasts [6]. Additionally, AZI was found to have an antioxidant effect. In rats treated with cisplatin, AZI has been shown to enhance antioxidant defenses against oxidative stress by raising levels of glutathione (GSH), superoxide dismutase (SOD), and glutathione-*S*-transferase (GST) [7].

This study proposes that AZI stimulates glutathione and superoxide dismutase and decreases important inflammatory and apoptotic agents, which in turn promotes neuronal survival and recovery in rats with TBI-induced neurochemical and neurobehavioral changes. By targeting oxidative stress and inflammation, AZI could potentially serve as a therapeutic agent in the management of TBI.

## 2. Results

The main objective of the present study is to determine the neuroprotective properties of AZT in rats subjected to TBI. Upon IP administration of AZI (150 mg/kg) on successive 28-day treatments, it reverses and improves the neurobehavioral as well as neurochemical parameters in a dose-dependent manner.

### 2.1. Effect of Azithromycin on Body Weight in Rat Model of TBI

**Figure 1:**
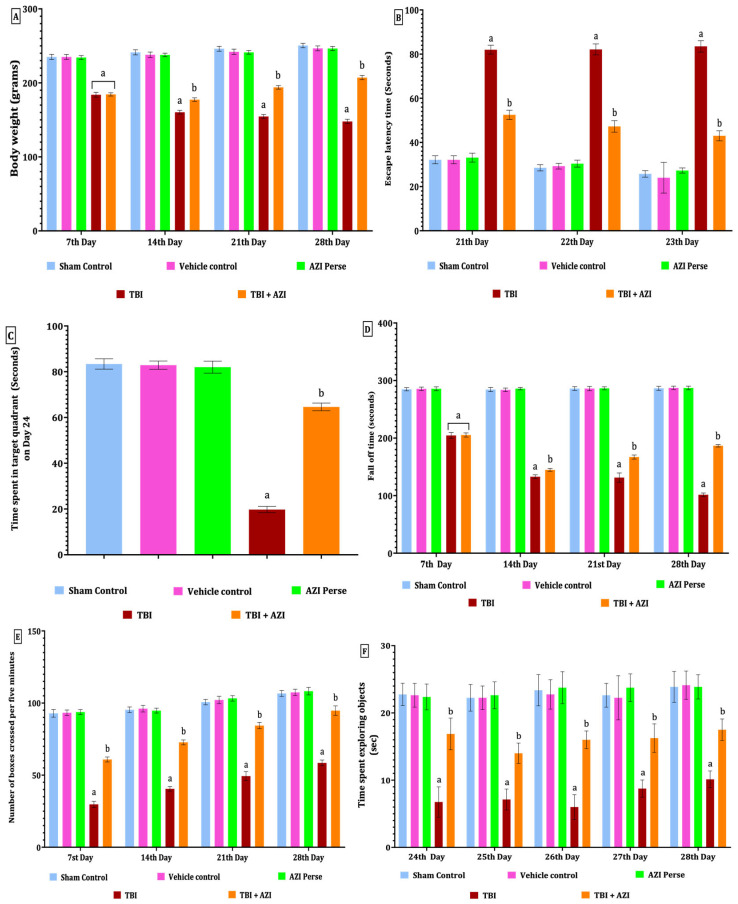
Therapeutic effectiveness of AZI in improving body weight and behavioral alterations in adult Wistar rat TBI model. Body weight (**A**), escape latency test (**B**), TSTQ (**C**), fall-off time on rotarod test (**D**), open field test (**E**), NORT (**F**). The statistical data was analyzed using two-way ANOVA with Bonferroni’s post hoc test for (**A**,**B**,**D**–**F**) and one-way ANOVA with Tukey’s post hoc test for (**C**). The results are presented as mean ± standard deviation (SD) for each experimental group, with a sample size of 8 Wistar rats per group, The statistical significance was indicated as *p* < 0.01. Comparing (a) versus sham, vehicle control, and AZI control and (b) versus TBI.

The body weights of animals were checked on days 1, 14, and 28 during the protocol schedule. As per the observation, there was a significant three-fold decrease in the body weight of the animals subjected to TBI, as compared to the sham control, vehicle control, and AZI groups; also, there was a significant improvement in the body weight of the TBI + AZI group, as compared to the TBI group (two-way ANOVA: F(4,35) = 1615; *p* < 0.01) (Figure 1A).

### 2.2. Effect of AZI on Neurobehavioral Assessment Task in Rat Model of TBI

#### 2.2.1. Effect of Azithromycin in Reversing Memory Impairment in Rat Model of TBI

The Morris water maze was performed to check memory deficits, based on two assessment parameters, i.e. escape latency (ELT) and time spent in target quadrant (TSTQ), and the same tasks were performed on days 21, 22, and 23 during the protocol schedule. As per the observation, results indicate that there was a significant increase in ELT in the TBI group, as compared to the sham control, vehicle control, and AZI groups, on successive days 21, 22, and 23; also, there was a significant decrease in the ELT in the TBI + AZI group, as compared to the TBI group, on successive day 21, 22 and 23. This indicates that AZI has a neuroprotective effect by improving and reversing memory impairment in the ELT task (Two-way ANOVA: F(4,35) = 1240; *p* < 0.01) (Figure 1B).

The TSTQ task was performed on day 24, exhibiting a significant decrease in the time spent in the target quadrant in the TBI group, as compared to the sham control, vehicle control, and AZI groups. However, the TBI + AZI group showed a significant increase in the TSTQ after long-term therapy, compared to the TBI group. This observation suggests an improvement in memory and spatial navigation tasks in the TBI + AZI-treated group [one-way ANOVA: F(2.583,18.08) = 1418; *p* < 0.01] (Figure 1C).

#### 2.2.2. Effect of Azithromycin on Neuromuscular Coordination in Rat Model of TBI

Rotarod was performed on days 7, 14, 21, and 28 during the protocol schedule to assess motor coordination. The cutoff time was 5 min to note the latency to fall of animals, denotes the neuromuscular coordination in rats. There was a significant decrease in the latency to fall in the TBI group throughout the whole protocol compared to the sham control, vehicle control, and AZI groups; also, there was a slightly significant decrease in latency to fall in the TBI + AZI group, as compared to the TBI group, on days 7, 14, and 21, and on day 28, there was significantly improved activity observed on the rotarod task by the TBI + AZI group, as compared to days 7 and 14. This result signifies the protective action of AZI, with improved neuromuscular coordination in the TBI animal model [two-way ANOVA: F(4,35) = 9816; *p* < 0.01] (Figure 1D).

#### 2.2.3. Effect of Azithromycin on Locomotor Activity in Rat Model of TBI

The open field test was conducted according to the protocol schedule on days 7, 14, 21, and 28. A cutoff time of 5 min was set to record the number of boxes crossed by the rat. In the TBI group, a significant decrease in locomotion movement activity was observed throughout the whole protocol schedule compared to the sham control, vehicle control, and AZI groups. In the TBI + AZI group, a significant increase in the number of boxes crossed per 5 min was observed on days 7 and 14, as compared to the TBI group. Additionally, locomotor activity continued to improve in the TBI + AZI group, with a further increase in the number of boxes crossed on days 21 and 28 compared to days 7 and 14. This result indicates that AZI improved and gradually restored the locomotor activity in the TBI animal model with long-term treatment [two-way ANOVA: F(4,35) = 2196; *p* < 0.01] (Figure 1E).

#### 2.2.4. Effect of Azithromycin on Memory in Rat Model of TBI

The novel object recognition test (NORT) was performed to assess memory on days 24, 25, 26, 27, and 28. Results indicate a memory deficit in the TBI group, exhibited as a significant decrease in the time spent exploring novel objects compared to the sham control, vehicle control, and AZI groups. In the TBI + AZI treated group, the animals’ memories were significantly improved after long-term AZI treatment, as compared to the TBI group on the successive 5 days of the behavioral NORT task. These findings suggest that AZI has a neuroprotective action in improving and restoring the recognition memory in the TBI animal model [two-way ANOVA: F(4,35) = 539.3; *p* < 0.01] (Figure 1F).

### 2.3. Histopathological Effects of AZI on TBI-Induced Damage in the Cortex, Hippocampus, and Striatum

**Figure 2:**
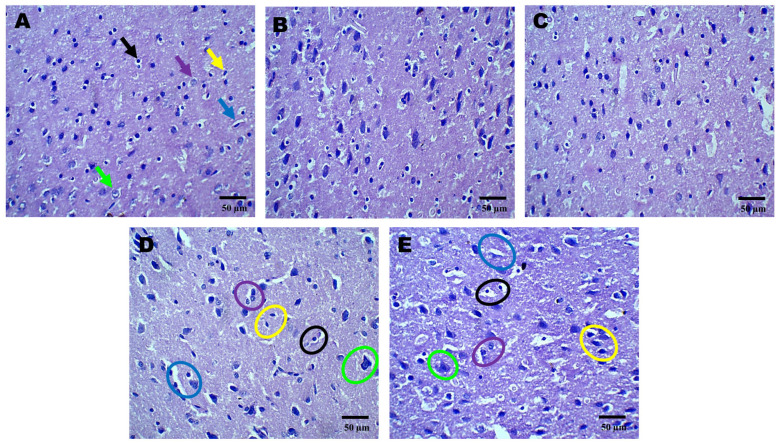
Neuroprotective effect shown by AZI reduces histopathological alterations (coronal frames –cortex) in experimental TBI rats. Histological analysis of cortical neurons stained with H&E in sham control (**A**), vehicle control (**B**), AZI (**C**), TBI (**D**), and TBI + AZI (**E**) groups. Panel (**A**) (sham control) shows normal cortical structure, with intact pyramidal cells (green arrow), astrocytes (purple arrow), microglia (yellow arrow), blood capillaries (blue arrow), oligodendrocytes (black arrows), with no visible structural abnormalities. Images of the TBI group show AZI’s neuroprotective effect, reducing structural damage and promoting recovery. The cortical gray matter was assessed for TBI-induced alterations. Color annotations highlight cell types: black circles (oligodendrocytes), purple circles (astrocytes), green circles (pyramidal cells), yellow circles (microglia), and blue circles (blood capillaries). In the TBI group (**D**), structural abnormalities were observed in oligodendrocytes (black circles), astrocytes (purple circles), and capillaries (blue circles). AZI treatment in the TBI + AZI group (**E**) showed restored structural integrity, with enhanced microglial activity, intact oligodendrocytes (black circles) and astrocytes (purple circles), and better-organized capillaries (blue circles). (Magnification = 40×; scale = 50 μM).

**Figure 3:**
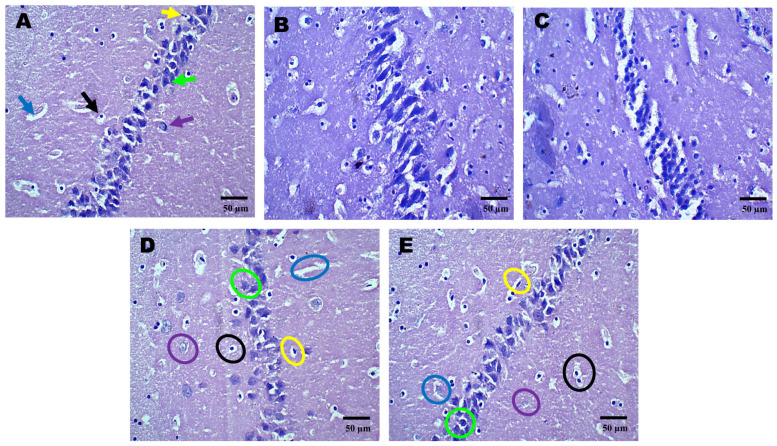
Neuroprotective effects shown by AZI reduce histopathological alterations (coronal frames–hippocampus) in experimental TBI rats. Histopathological analysis of the hippocampal region stained with H&E in sham control (**A**), vehicle control (**B**), AZI (**C**), TBI (**D**), and TBI + AZI (**E**) groups. Panel (**A**) shows the normal hippocampal structure with the alveus (white matter) on the inner side and the fimbria (gray matter) on the outer side. Pyramidal cells (green arrow), astrocytes (purple arrow), microglia (yellow arrow), blood capillaries (blue arrow), oligodendrocytes (black arrows), all appear intact. In the vehicle control (**B**) and AZI per se (**C**) groups, oligodendrocytes (black circles) and astrocytes (purple circles) remain viable. However, in the TBI group (**D**), cellular deterioration is evident, with deformed oligodendrocytes (black circles) and astrocytes (purple circles). In the TBI + AZI group (**D**), histological improvements are observed, with restored pyramidal cells (green circles), oligodendrocytes (black circles), and astrocytes (purple circles), indicating AZI’s neuroprotective effects. (Microglia are marked in yellow circles and blood capillaries in blue circles). (Magnification = 40×; scale = 50 μM).

**Figure 4:**
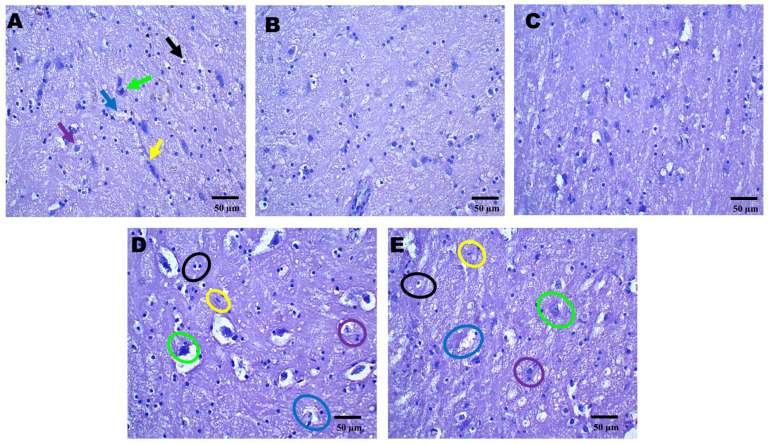
Neuroprotective effects shown by AZI reduce histopathological alterations (coronal frames–striatum) in experimental TBI rats. Histological analysis of the striatum stained with H&E across sham control (**A**), vehicle control (**B**), AZI (**C**), TBI (**D**), and TBI + AZI (**E**) groups. Panel (**A**) shows the normal structure of the dorsal striatum, with organized white matter internally and gray matter externally. The molecular layer contains neuronal nuclei, with oligodendrocytes (black arrow) in the intermediate layer and astrocytes (purple arrow) within the extracellular matrix, pyramidal cells (green arrow), microglia (yellow arrow), and blood capillaries (blue arrow). In the vehicle control (**B**) and AZI per se (**C**) groups, cellular alignment remains intact. However, in the TBI group (**D**), irregularly shaped oligodendrocytes (black circles) and impaired astrocytes (purple circles) indicate cellular dysfunction. AZI treatment in the TBI + AZI group (**E**) shows histological restoration, with increased density of oligodendrocytes (black circles) and astrocytes (purple circles), suggesting AZI’s therapeutic effect on cellular integrity and tissue recovery. Additional annotations include microglia (yellow circles), pyramidal cells (green circles) and blood capillaries (blue circles). (Magnification = 40×; scale = 50 μM).

Histopathological assessment of sections from different brain regions was performed. Histopathological alterations were found in the cortex, hippocampus, and striatum of the TBI group compared to the sham group. In the cortex, oligodendrocytes and astrocytes appeared compromised, with signs of deformation and clustering (Figure 2D). These cells showed disorganized arrangements, and there was visible cellular debris, indicating cellular injury. Similarly, structural alternations were found in the hippocampus (Figure 3D) and striatum (Figure 4D). On the other hand, animals treated with AZI after TBI showed marked improvements in cortical (Figure 2E), hippocampal (Figure 3E), and striatal (Figure 4E) structures compared to the TBI group. Oligodendrocytes and astrocytes displayed more typical morphology, with reduced signs of clustering and deformation, indicating that AZI may help restore cellular integrity.

### 2.4. Effect of AZI on Oxidative Stress Parameters in Rat Model of TBI

**Figure 5:**
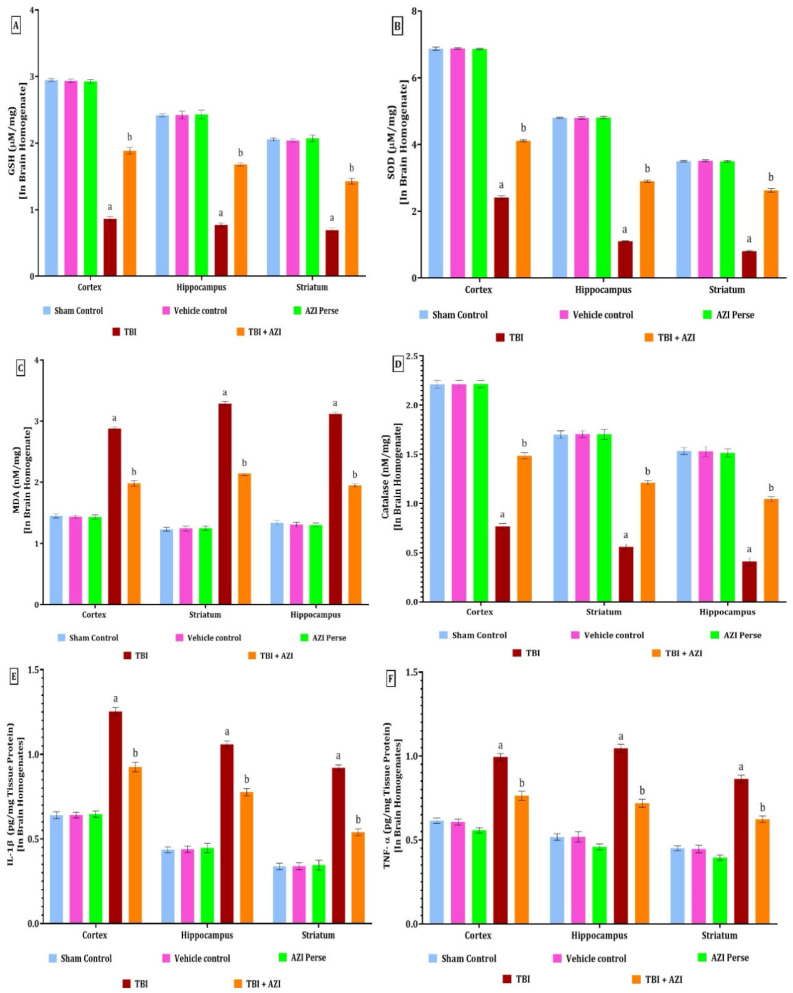
Therapeutic efficacy of AZI in modulation of antioxidant levels and reducing the inflammatory markers in brain homogenate of rat model of TBI. GSH (**A**), SOD (**B**), MDA (**C**), catalase (**D**), IL-1β (**E**), TNF-α (**F**). The statistical data was presented using the one-way ANOVA analysis with Tukey’s test. The values were reported as the mean ± standard deviation (SD) for each experimental group, with a sample size of 8 Wistar rats per group. The statistical significance was indicated as *p* < 0.01. Comparing (a) versus sham, vehicle control, and AZI control and (b) versus TBI.

The levels of GSH, SOD, MDA, and catalase were estimated using the ELISA technique at the end of the protocol schedule. The antioxidant levels were estimated in the cortex, striatum, and hippocampus regions of the rat brain homogenate. MDA is a well-known biomarker of lipid peroxidation and oxidative stress. The malondialdehyde (MDA) levels were significantly elevated in the TBI group in the cortex, striatum, and hippocampus regions of the rat brain homogenate, as compared to the sham control, vehicle control, and AZI groups, and there was a significant decrease in the MDA level in the TBI + AZI group, as compared to TBI group, in the cortex, striatum, and hippocampus regions of the rat brain homogenate (Figure 5C). The statistical significance of differences was assessed by performing a one-way analysis of variance (ANOVA) on the brain homogenate for estimation of MDA collected from several brain areas, including the cortex [one-way ANOVA: F(4,28) = 2607; *p* < 0.01], hippocampus [one-way ANOVA: F(4,28) = 3909; *p* < 0.01], and striatum [one-way ANOVA: F(4,28) = 5087; *p* < 0.01].

In the TBI group, the levels of GSH, SOD, and catalase were significantly decreased in the cortex, striatum, and hippocampus regions, as compared to the sham control, vehicle control, and AZI groups. The decreased level of GSH was significantly increased in the TBI + AZI group, as compared to the TBI group, in the cortex, striatum, and hippocampus regions. The results showed that AZI modulates the antioxidant level and oxidative stress markers in the rat model of TBI (Figure 5A,B,D).

The statistical significance of differences was assessed by performing a one-way analysis of variance (ANOVA) on the brain homogenate for the estimation of GSH collected from several brain areas, including the cortex [one-way ANOVA: F(4,28) = 5813; *p* < 0.01], hippocampus [one-way ANOVA: F(4,28) = 1939; *p* < 0.01], and striatum [one-way ANOVA: F(4,28) = 2108; *p* < 0.01]. The statistical significance of differences was assessed by performing a one-way analysis of variance (ANOVA) on the brain homogenate for the estimation of SOD collected from several brain areas, including the cortex [one-way ANOVA: F(4,28) = 20,179; *p* < 0.01], hippocampus [one-way ANOVA: F(4,28) = 31492; *p* < 0.01], and striatum [one-way ANOVA: F(4,28) = 9942; *p* < 0.01]. The statistical significance of differences was assessed by performing a one-way analysis of variance (ANOVA) on the brain homogenate for estimation of catalase collected from several brain areas, including the cortex [one-way ANOVA: F(4,28) = 2549; *p* < 0.01], hippocampus [one-way ANOVA: F(4,28) = 1105; *p* < 0.01], and striatum [one-way ANOVA: F(4,28) = 1769; *p* <0.01].

### 2.5. Effect of AZI on Inflammatory Markers in Rat Model of TBI

The levels of IL-1β and TNF-α in the cortex, hippocampus, and striatum regions were estimated using the ELISA technique at the end of the protocol schedule. The levels of IL-1β and TNF-α in the cortex, hippocampus, and striatum were significantly elevated in the TBI group, as compared to the sham control, vehicle control, and AZI groups. The elevated levels of IL-1β and TNF-α in the cortex and striatum were significantly decreased in the TBI + AZI group, as compared to the TBI group, in the cortex, striatum and hippocampus regions (Figure 5E,F). The statistical significance of differences was assessed by performing a one-way analysis of variance (ANOVA) on the brain homogenate for the estimation of IL-1β collected from several brain areas, including the cortex [one-way ANOVA: F(4,28) = 1192; *p* < 0.01], hippocampus [one-way ANOVA: F(4,28) = 1891; *p* < 0.01], and striatum [one-way ANOVA: F(4,28) = 1507; *p* < 0.01]. The statistical significance of differences was assessed by performing a one-way analysis of variance (ANOVA) on the brain homogenate for the estimation of TNF-α collected from several brain areas, including the cortex [one-way ANOVA: F(4,28) = 617.3; *p* < 0.01], hippocampus [one-way ANOVA: F(4,28) = 758.9; *p* < 0.01], and striatum [one-way ANOVA: F(4,28) = 921.4; *p* < 0.01]. 

### 2.6. Effect of AZI on Apoptotic Markers in Rat Model of TBI

The levels of caspase-3, Bax, and Bcl-2 in the cortex, hippocampus, and striatum were estimated using the ELISA method at the end of the protocol schedule. Caspase-3 is a key effector in the apoptotic pathway, and its activation is closely associated with neuronal cell death following TBI. Bax (Bcl-2-associated X protein) is a pro-apoptotic member of the Bcl-2 protein family, and its increased expression is associated with neuronal apoptosis following TBI.

**Figure 6:**
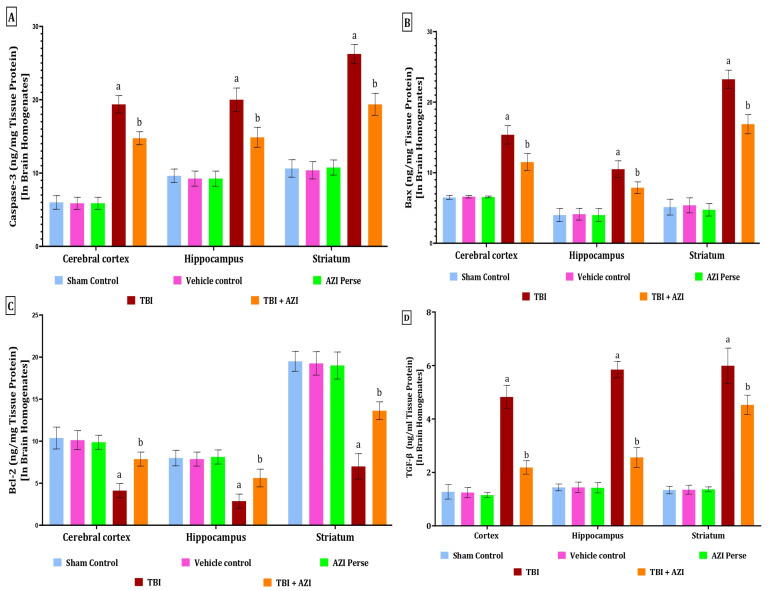
Therapeutic efficacy of AZI in modulation of apoptotic markers and *TGF-β* in brain homogenate of rat model of TBI. Caspase-3 (**A**), Bax (**B**), Bcl-2 (**C**), TGF- β (**D**). The statistical data was presented using the one-way ANOVA analysis with Tukey’s test. The values were reported as the mean ± standard deviation (SD) for each experimental group, with a sample size of 8 Wistar rats per group. The statistical significance was indicated as *p* < 0.01. Comparing (a) versus sham, vehicle control, and AZI control and (b) versus TBI.

The levels of caspase-3 and Bax in the cortex, hippocampus, and striatum were significantly increased in the TBI group, as compared to the sham control, vehicle control, and AZI groups. The increased level of caspase-3 and Bax in the cortex, hippocampus, and triatum was significantly reduced in the TBI + AZI treatment group, as compared to the TBI group. The statistical significance of differences was assessed by performing a one-way analysis of variance (ANOVA) on the brain homogenate for the estimation of caspase-3 collected from several brain areas, including the cortex [one-way ANOVA: F(4,28) = 322.4; *p* < 0.01], hippocampus [one-way ANOVA: F(4,28) = 155.1; *p* < 0.01], and striatum [one-way ANOVA: F(4,28) = 224.6; *p* < 0.01] (Figure 6A). The statistical significance of differences was assessed by performing a one-way analysis of variance (ANOVA) on the brain homogenate for the estimation of Bax collected from several brain areas, including the cortex [one-way ANOVA: F(4,28) = 173.7; *p* < 0.01], hippocampus [one-way ANOVA: F(4,28) = 81.70; *p* < 0.01], and striatum [one-way ANOVA: F(4,28) = 479.4; *p* < 0.01] (Figure 6B).

The levels of Bcl-2 in the cortex, hippocampus, and striatum were significantly decreased in the TBI group, as compared to the sham control, vehicle control, and AZI groups. The decreased levels of Bcl-2 in the cortex, hippocampus, and striatum were significantly increased and improved in the TBI + AZI treatment group after 28 days of AZI treatment. These results indicate the neuroprotective action produced by AZI by modulating the apoptotic markers in a significant manner. The statistical significance of differences was assessed by performing a one-way analysis of variance (ANOVA) on the brain homogenate for the estimation of Bcl-2 collected from several brain areas, including the cortex [one-way ANOVA: F(4,28) = 50.46; *p* < 0.01], hippocampus [one-way ANOVA: F(4,28) = 45.08; *p* < 0.01], and striatum [one-way ANOVA: F(4,28) = 190.9; *p* < 0.01] (Figure 6C).

### 2.7. Effect of AZI on TGF- β in Rat Model of TBI

The level of TGF-β as a neurofilament (NEFL) marker was estimated in the cortex, striatum, and hippocampus regions of the rat brain homogenate using the ELISA technique at the end of the protocol schedule. TGF-β (transforming growth factor β-1) is a key cytokine that plays a crucial role in the inflammatory response and tissue-repair processes following TBI. The level of TGF-β was significantly elevated in the TBI group in the cortex, striatum, and hippocampus regions of the rat brain homogenate, as compared to the sham, vehicle control, and AZI groups. The elevated levels of TGF-β in the cortex, striatum, and hippocampus regions of the rat brain homogenate were significantly decreased in the TBI + AZI treatment group, as compared to TBI group. The statistical significance of differences was determined by performing a one-way analysis of variance (ANOVA) on the brain homogenate collected from several parts of the brain, particularly the cortex [one-way ANOVA: F(4,28) = 256; *p* < 0.01], hippocampus [one-way ANOVA: F(4,28) = 480.9; *p* < 0.01], and striatum [one-way ANOVA: F(4,28) = 277.9; *p* < 0.01] (Figure 6D).

**Figure 7:**
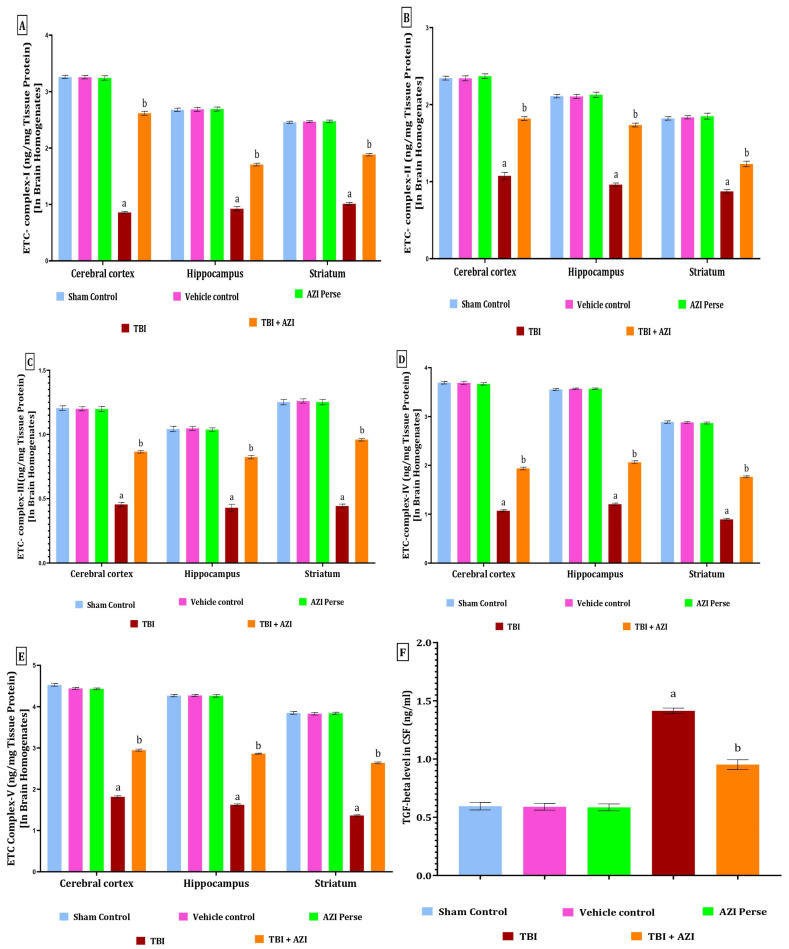
Therapeutic efficacy of AZI in restoring cellular and molecular target protein levels in brain homogenate of experimental rat model of TBI. ETC complex I (**A**), complex II (**B**), complex III (**C**), complex IV (**D**), complex V (**E**), and TGF-beta (**F**). The statistical data was presented using the one-way ANOVA analysis with Tukey’s test. The values were reported as the mean ± standard deviation (SD) for each experimental group, with a sample size of 8 Wistar rats per group. The statistical significance was indicated as *p* < 0.01. Comparing (a) versus sham, vehicle control, and AZI control and (b) versus TBI.

The level of TGF-β was estimated in the CSF as well, using the ELISA technique at the end of the protocol schedule. The level of TGF-β was significantly elevated in the TBI group in the CSF of the rat, as compared to the sham, vehicle control, and AZI per se groups. The elevated level of TGF-β in the CSF of the rat was significantly decreased in the TBI + AZI-150 mg treatment group, as compared to the TBI group. The statistical significance of differences was determined by performing a one-way analysis of variance (ANOVA) on the CSF [one-way ANOVA: F(4,28) = 256; *p* < 0.01] (Figure 7F).

### 2.8. Effect of AZI on Mitochondrial Complexes in Rat Model of TBI

The levels of mitochondrial complexes (complex I, II, III, IV, and V) in the cortex, hippocampus, and striatum regions were estimated using ELISA method at the end of 28 days protocol schedule. The levels of mitochondrial complex I, II, and V in the cortex, hippocampus, and striatum were significantly two-fold decreased in the TBI group, as compared to the sham control, vehicle control, and AZI groups. The reduced levels of mitochondrial complex I, II, and V in the cortex, hippocampus, and striatum were significantly increased in the TBI + AZI treatment group after 21 days of therapy, as compared to the TBI group. The biological sample used in this study includes the cerebral cortex, hippocampus, and striatum. The statistical significance of the ETC complex I was assessed using a one-way analysis of variance (ANOVA), including the cortex [one-way ANOVA: F(4,28) = 9832; *p* < 0.01], hippocampus [one-way ANOVA: F(4,28) = 5291; *p* < 0.01], striatum [one-way ANOVA: F(4,28) =7759; *p* < 0.01], complex II in cortex [one-way ANOVA: F(4,28) = 2240; *p* < 0.01], hippocampus [one-way ANOVA: F(4,28) = 4745; *p* < 0.01], striatum [one-way ANOVA: F(4,28) = 1881; *p* < 0.01], complex III in cortex [one-way ANOVA: F(4,28) = 3036; *p* < 0.01], hippocampus [one–way ANOVA: F(4,28) = 1582; *p* < 0.01], striatum [one–way ANOVA: F(4,28) = 3354; *p* < 0.01], complex IV in cortex [one-way ANOVA: F(4,28) = 16,431; *p* < 0.01], hippocampus [one-way ANOVA: F(4,28) = 23,966; *p* < 0.01], striatum [one-way ANOVA: F(4,28) = 13,358; *p* < 0.01], complex V in cortex [one-way ANOVA: F(4,28) = 18,571; *p* < 0.01], hippocampus [one–way ANOVA: F(4,28) = 17,026; *p* < 0.01], striatum [one-way ANOVA: F(4,28) = 32,195; *p* < 0.01] (Figure 7A–E).

## 3. Discussion

The neuroprotective role of AZI in TBI represents a significant preclinical assessment of AZI that supports its therapeutic potential. Recent research has demonstrated that AZI not only possesses antimicrobial properties but also exhibits neuroprotective effects, particularly in the context of ischemic conditions. This study demonstrated that AZI administration significantly improved behavioral deficits, reduced inflammation, and attenuated neuronal loss following TBI.

AZI has been shown to reduce inflammation in models of brain injury. In neonatal rats subjected to hypoxic–ischemic brain injury, AZI was shown to significantly improve sensorimotor function and reduce tissue damage, indicating its potential as a neuroprotective agent in inflammatory events associated with TBI [8]. AZI’s ability to modulate inflammatory responses is crucial, as systemic and local inflammation can worsen brain injury.

The behavioral assessments including escape latency, TSTQ, rotarod, open field tests, and NORT conducted in this study revealed a significant improvement and restoration in memory, motor function, neuromuscular strength and cognitive performance in AZI-treated rats compared to the TBI group. TBI is well-documented to result in deteriorations of memory and motor and cognitive functions, as shown in previous studies, also report the neuroprotective effects of AZI in various neurological disorders, including stroke, PD, and AD [9,10]. The observed behavioral benefits in this study and in the literature are in agreement regarding the ability of AZI to reduce neuronal damage and inflammation, thereby promoting functional recovery.

The findings from preclinical studies support the hypothesis that AZI could be repurposed as a therapeutic agent for TBI management. Given its established safety profile and anti-inflammatory properties, AZI presents a promising option for the management of TBI, particularly in patients with concurrent infections or inflammatory conditions [8,11].

In addition, TBI induces excessive production of reactive oxygen species (ROS) due to mitochondrial dysfunction. Electron leakage from the ETC can generate ROS, which leads to further mitochondrial damage and neuronal apoptosis. Elevated MDA levels after TBI was shown in previous studies, where increased oxidative stress was linked to elevated levels of MDA, indicating lipid peroxidation in TBI-subjected rats, while AZI treatment significantly reduced MDA levels [8,12].

As for GSH levels, a significant decrease is a common outcome following TBI. In a study by Koza and Daniel [13], GSH levels were quantified in the hippocampi of experimental rats after a unilateral moderate cortical contusion model of TBI. A time-dependent decrease in GSH levels was evident, with significant reductions observed as early as 3 h post-injury, reaching the lowest values at 24–48 h post-TBI compared to sham controls [14]. Another study found that GSH levels in the cerebrospinal fluid of infants and children with severe TBI were significantly lower from day 1 post-injury until day 7, when compared to healthy controls [15].

Consistent with these findings, a study examining the effects of TBI on oxidative stress markers reported a significant decrease in SOD activity in the brain tissues of TBI rats. This decline is associated with increased oxidative stress and neuronal damage following injury. The study highlighted that reduction in SOD activity contributes to the accumulation of ROS, exacerbating secondary injury mechanisms post-TBI [16]. The decrease in SOD levels post-TBI has been correlated with impaired cognitive functions and increased behavioral deficits, highlighting the importance of maintaining antioxidant enzyme activity for neuronal health and recovery after brain injury [17,18]. As per the present study, the decline in GSH, SOD, and catalase levels in TBI-subjected rats was partially reversed by AZI treatment.

The neuroprotective effect of AZI is exerted through immunomodulatory actions, particularly by promoting the polarization of macrophages towards an anti-inflammatory (M2) phenotype. This shift helps in reducing the neurotoxic effects associated with pro-inflammatory (M1) macrophages, which are prevalent in TBI [18]. By altering macrophage activation, AZI can mitigate secondary injury processes following TBI. Consistently, this study reported an increased level of inflammatory markers including TNF-α and IL-1β in TBI rats that was significantly reduced after AZI administration, which supports the neuroprotective effect of AZI.

Mitochondria have a major role in apoptosis, through the release of pro-apoptotic factors such as cytochrome c. TBI can result in elevated levels of pro-apoptotic proteins, such as Bax, alongside decreased levels of anti-apoptotic proteins including Bcl-2. This shift promotes cell death pathways that are exacerbated by mitochondrial dysfunction [19,20].

A study utilizing the modified free-falling body impact method to induce TBI in rats has reported that the expression of Bax was significantly higher in the TBI group compared to sham controls. Gene-expression analysis of Bax indicated that the level of Bax expression was markedly elevated in TBI rats, suggesting that brain injury leads to increased apoptosis of neuronal cells due to elevated Bax levels [16,21]. Overexpression of Bax has been linked to increased neuronal cell death following TBI. In a study examining the effects of TBI on neuronal apoptosis, it was found that increased Bax levels were associated with a reduction in Bcl-2 levels, further promoting apoptosis in affected neurons [16]. The role of Bax in apoptosis following TBI is further supported by findings that link its activation to mitochondrial dysfunction and cytochrome c release. Increased levels of Bax can lead to mitochondrial outer-membrane permeabilization, facilitating apoptotic processes and contributing to secondary injury mechanisms following TBI [16].

Recent research indicated that caspase-3 activation occurs in the superficial cortical areas and thalamus of rats as early as 1 h post-TBI. This activation is associated with both intrinsic and extrinsic apoptotic pathways, contributing to secondary brain injury mechanisms following the initial trauma [22,23]. Elevated levels of caspase-3 have been correlated with the severity of TBI, which is backed by our study findings, in which an increase in the level of caspase-3 in brain homogenate was reported. In animal models where a more severe injury was induced, such as with higher impact forces, the levels of activated caspase-3 were markedly increased, suggesting a direct relationship between injury severity and apoptotic signaling [23]. The activation of caspase-3 has been linked to delayed neuronal death, which is associated with neurological deficits and poor outcomes following TBI. It has been shown that the inhibition of caspase-3 activity can lead to reduced neuronal loss and improved functional recovery in TBI models, emphasizing its role as a critical mediator of cell death [16,24]. The activation of caspase-3 is also involved in the inflammatory response following TBI. Inflammatory caspases, including caspase-3, contribute to the neuroinflammatory processes that exacerbate neuronal injury and promote apoptotic cell death after TBI [16,24]. In this study, it was evident that increased Bax and caspase-3 levels in TBI rats were significantly reduced after AZI treatment.

In rats subjected to TBI, TGF-β levels significantly increased. A previous study reported that TGF-β mRNA expression was markedly increased in the cerebral cortex of rats after a penetrating brain injury, showing strong expression four days post-injury. This suggests a robust response of TGF-β signaling in the injured brain, likely contributing to neuroinflammatory processes and tissue repair mechanisms [25]. The elevation of TGF-β following brain injury is associated with both pro-inflammatory and reparative responses. It has been shown to stimulate nerve growth factor production and modulate astrocyte proliferation, which is critical for scar formation and recovery processes in the injured brain [20,25]. Studies have reported significant increases in TGF-β levels in brain injury as compared to baseline measurements, as confirmed by results of the current study. After AZI treatment, TGF-β gene expression levels in brain homogenate were partially restored to normal levels.

Mitochondrial complexes were also studied in this investigation to establish the pathophysiological relevance of AZI in TBI. The dysfunction of mitochondrial complexes contributes to energy deficits, oxidative stress, and neuronal apoptosis [26]. In TBI, mitochondrial respiration is often impaired, leading to decreased ATP levels, which are critical for neuronal survival and function. Studies have shown that TBI results in reduced activity of mitochondrial complexes, particularly complex I and IV, contributing to energy deficits in the injured brain [19,27]. In another study, complex I activity has been reported to decline significantly post-injury, correlating with increased oxidative stress markers and neuronal damage, as supported by our study findings [16,17]. The differential regulation of the levels of mitochondrial complexes I–V in this study provided additional evidence in the effect of TBI on mitochondrial complexes and the usefulness of AZI in the management of TBI.

In a transient focal ischemia rat model, AZI administration was associated with a significant reduction in brain infarct damage and neurological deficits. This effect was linked to the elevation of signal transducer and activator of transcription 3 (STAT3) phosphorylation, which functions in cellular survival and inflammatory response modulation [12]. The timing of AZI administration post-injury further emphasizes its potential as an acute treatment option for TBI. Research also indicated that a single dose of AZI can provide long-lasting neuroprotection, with benefits observed up to seven days after administration. This suggests that AZI could be effective even when treatment is initiated after the onset of brain injury, which is critical in clinical settings where immediate treatment may not be feasible [12]. The administration of AZI at a dose of 150 mg/kg has been shown to significantly reduce brain infarct damage and improve neurological outcomes following transient focal cerebral ischemia and in sciatic nerve injury in vivo [28]. This effect is notable, given that the effective dose (ED50) of AZI in this context is substantially lower than its bactericidal doses, indicating a favorable therapeutic window for its application in neuroprotection. Specifically, AZI was effective even when administered up to 4.5 h post-injury, with neuroprotective effects lasting up to seven days post-treatment [12]. In order to obtain consistent results between replicates, in addition to dose adjustment, the sample size for each group was *N* = 6, with each measurement taken three times to ensure accuracy and reproducibility. For behavioral studies, each animal was tested three times, and the results were averaged to represent one replicate (n). Additionally, the consistency was enhanced by carefully controlled experimental conditions, including standardized procedures for animal handling, protocols, treatment administration, and all experimental environments.

The neuroprotective effects of AZI in TBI can be mediated through several mechanisms. One potential mechanism involves the inhibition of inflammatory pathways. AZI has been shown to reduce the expression of inflammatory cytokines and chemokines, which can contribute to secondary brain injury [3]. Additionally, AZI may exert neuroprotective effects by modulating the activity of neurotransmitters and signaling pathways involved in neuronal survival and plasticity [16,29].

The underlying mechanisms of the neuroprotective effects of AZI were linked to its ability to modulate inflammatory responses. Previous studies indicated that AZI promotes the phosphorylation of signal transducer and activator of transcription 3 (STAT3) in various cell types within the ischemic brain, including neurons and astrocytes. STAT3 phosphorylation is associated with the activation of M2 macrophages, which are known to play a role in tissue repair and anti-inflammatory processes. Such immunomodulatory properties are consistent with findings from other studies that highlight the role of AZI in promoting beneficial macrophage polarization and reducing neuroinflammation [12,30].

The potential to repurpose AZI as a neuroprotective agent in human TBI cases is supported by its existing safety profile in humans, where it is commonly used to treat respiratory infections. However, while AZI has shown promise in preclinical studies, translating these findings to human clinical practice will require further investigation, including well-designed clinical trials to assess the efficacy and safety of AZI in TBI patients [31].

Moreover, the exploration of AZI’s neuroprotective effects in other conditions, such as stroke and retinal ischemia, reinforces its potential as a versatile therapeutic agent. The ability to mitigate neuronal damage through mechanisms that involve immune modulation positions AZI as a candidate for further research into its role in neuroprotection across various neurological disorders [12].

### Limitations and Future Directions

While this study provided evidence for the neuroprotective effects of AZI in TBI, it is important to acknowledge certain limitations. The use of a rat model may not fully capture the complexity of human TBI, and additional studies are needed to validate these findings in humans.

Future research should also focus on investigating the mechanisms of action of AZI in TBI, evaluating its efficacy in human clinical trials, and exploring potential combination therapies with other neuroprotective agents. Additionally, studies are needed to assess the long-term safety and tolerability of AZI in TBI patients.

## 4. Materials and Methods

### 4.1. Animals

Healthy male Wistar rats of 4–5 months were provided by the Central Animal House of the ISF College of Pharmacy in Moga, Punjab, under registration number 816/PO/ReBiBt/S/04/CCSEA. The Institutional Animal Ethics Committee (IAEC) accepted the ISFCP/IAEC/CCSEA/Meeting No: 04/ Protocol No-22 in the meeting held in 4/11/2023, following Indian government requirements. Ethical approval was also obtained from the College of Pharmacy, Taibah University, research ethics committee (COPTU-REC-117-2024812). Animals were maintained under standard laboratory conditions of temperature (21 ± 1°C), and typical husbandry environment, with an unlimited supply of food and water and a 12 h reverse light cycle. The protocol was done according to the guidelines of CCSEA.

### 4.2. Experimental Animal Grouping

Experimental animals were divided randomly into five groups. Group 1 animals were shammed against TBI by receiving minor cuts and the same dietary intake as that of the TBI and treatment groups. Group 2 animals were administered saline only to establish the vehicle’s inertness. The third group received AZI (Sun Pharma, Mumbai, India) intraperitoneal (IP) at 150 mg/kg [28] to check for any toxic or alternate effects of AZI biochemically and physiologically without subjecting the animals to TBI. Animals of group four were subjected to TBI through the weight drop method to check the pathological alteration of the disease. To assess the therapeutic benefits of AZI in TBI, a dose of 150 mg/kg was given IP to the fifth group with TBI induction.

### 4.3. Experimental Model

Traumatic brain injury was induced in rats using a weight drop method, which is a validated and established model as reported previously [7,32]. Initially, rats were anesthetized, and the fur on their heads was shaved to expose the skull and locate coronal and lambdoid sutures. A metallic disc was then securely attached to the identified area. The TBI was inflicted by dropping a 450 g metallic disc onto the rat’s head through a guiding pipe, which directly led to the weight drop-induced damage to the central part of the skull [17]. A foam pad measuring 10 cm in thickness was placed underneath the animal to mitigate the risk of secondary impacts from the weight drop. Afterwards, the metallic disc was removed, and the skin was sutured. In post-operative treatment, rats were housed individually in polyacrylic cages containing warm cloths and husks, and special care was performed till spontaneous movement was restored. Milk and glucose water were held in the cages for 2–3 days following surgery. The rats were monitored for feeding behavior, water intake, and overall activity for 14 days. Treatment commenced on the first day and continued until day 28 (Figure 1).

### 4.4. Experimental Protocol Schedule

**Figure 8:**
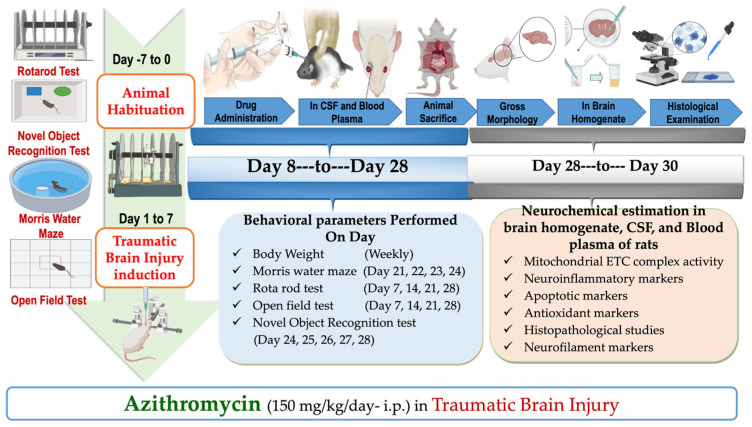
Experimental protocol schedule.

Before the induction of TBI, rats were habituated on the Morris water maze, rotarod, novel object recognition apparatus, and open field. After training, rats were randomly divided into 5 groups: sham (group 1), vehicle (group 2), AZI without TBI (group 3), TBI only (group 4), and AZI with TBI (group 5), comprising 6 animals per group. During the first week of the protocol, TBI was performed in the assigned groups, whereas minor cut-mimicking surgery was performed on animals of the sham group. AZI and normal saline were administered to the AZI without TBI and vehicle groups, respectively. Post-injury, the rats were monitored for 14 days, during which they underwent various behavioral assessments, including Morris water maze, novel object recognition test, open field to evaluate spatial learning and memory, and the rotarod test to assess motor coordination and balance, which were conducted at regular intervals (Figure 8). After the 28-day treatment period, rats were humanely euthanized, and brains were collected for analysis. Neurochemical estimation in brain homogenate, including the assessment of mitochondrial electron transport chain (ETC) complexes, specifically complex I, complex II, complex III, complex IV, and complex V. The assays also involved measuring apoptotic markers such as caspase-3, Bax (Bcl-2-associated X protein), and Bcl-2 (B-cell lymphoma 2), in addition to antioxidant markers including superoxide dismutase (SOD), reduced glutathione (GSH), malondialdehyde (MDA), and catalase. Neuroinflammatory markers were assessed, including βIL-1β, TNF-α, IL-6, and transforming growth factor-β (TGF-β).

### 4.5. Neurobehavioral Experiments

#### 4.5.1. Rotarod

Animal balance, motor planning, and muscle coordination were assessed using a rotarod apparatus acquired from the INCO group of companies (Ambala, India). The maximum rotational speed was set to 15 rpm, the animal’s latency to fall was recorded on days 1, 8, 22, and 33, and latency time was evaluated for 5 min [14].

#### 4.5.2. Morris Water Maze

The Morris water maze (MWM) was employed to evaluate spatial memory and cognitive deficits in experimental animals. Rats were allowed to swim freely in an opaque-filled circular tank and were familiarized with reaching a hidden platform located in one quadrant. After completing the training protocol, escape latency time (ELT) to reach the hidden platform was measured on days 31, 32, 33, and 34, serving as a determinant of memory formation in the rats. On day 35, the time spent in the target quadrant (TSTQ) zone was examined for 120 s to assess relative memory consolidation [33].

#### 4.5.3. Novel Object Recognition Test

The novel object recognition test (NORT) was conducted to evaluate recognition memory in rats. This test involved placing the animals in an arena containing two identical objects during the training phase. After a retention interval of several hours, one object was replaced with a novel object during the testing phase. The time spent exploring each object was recorded, with a preference for the novel object indicating intact recognition memory. The test was performed on days 36 and 37 to assess cognitive function and memory retention [34].

#### 4.5.4. Open Field Test

The open field test (OFT) was utilized to assess general locomotor activity and anxiety-like behavior in rats. Each rat was placed in a square arena divided into central and peripheral zones. The total distance traveled and time spent in the center versus the periphery were recorded for 10 min. Increased time spent in the center zone indicated reduced anxiety level, while decreased activity indicated cognitive impairment or anxiety-related behavior [35].

#### 4.5.5. Body Weight

Body weight is a general parameter used in assessing the health status of experimental rats, particularly in studies involving disease models like TBI. Throughout the experimental protocol, each rat’s body weight was measured weekly in grams to monitor any changes that may have indicated improvements or deteriorations in their health condition [36].

### 4.6. Biochemical and Neurochemical Investigations

#### 4.6.1. Biochemical Assays

To assess lipid peroxidation, the concentration of MDA was quantified in brain homogenates of cortex, hippocampus, and striatum [37]. Colorimetric measurements were performed, recording the absorbance of MDA at 532 nm with a UV-1700 spectrophotometer (Shimadzu, Tokyo, Japan) and expressed in nanomoles per milligram of protein. Additionally, nitrite levels were determined using Griess reagent, which contains 0.1% *N*-(1-naphthyl) ethylenediamine dihydrochloride, 1% sulfanilamide, and 2.5% phosphoric acid [38]. Equal volumes of the Griess reagent and tissue supernatant from the striatum and cortex were mixed at room temperature for 10 min, and the absorbance was measured at 540 nm. The results were expressed in micrograms per milliliter. The concentration of reduced glutathione in tissue homogenates from the striatum and cortex was measured. Tissue supernatant was combined with 4% sulfosalicylic acid at 4 °C until precipitation occurred, followed by centrifugation at 12,000× *g* for 15 min to separate the supernatant. This supernatant was then diluted with a phosphate buffer (0.1 M), and the development of a yellow color indicated the presence of glutathione. The absorbance was measured at 412 nm using a UV spectrophotometer, and the results were expressed as micromoles per milligram of protein [39].

#### 4.6.2. ELISA Assay

Homogenates from brain tissues, cerebrospinal fluid (CSF), and blood plasma were mixed with phosphate-buffered saline (PBS) and then centrifuged at 10,000 rpm for 5 min at 4 °C. Supernatants were collected and assayed using ELISA kits by following the manufacturer’s guidelines. Sample quantification was performed by comparing the results to standard curves. Each sample in each group was measured in duplicate or triplicate to ensure accuracy. The ELISA kits were employed in the quantification of apoptotic marker bcl-2 (KLR1880; Krishgen biosystem, Mumbai, India), Bax (KLR0034; Krishgen biosystem, Mumbai, India), and caspase-3 (KLR1648; Krishgen biosystem, Mumbai, India). ELISA kits for the evaluation of neuroinflammatory cytokines of TNF- alpha (KB1145; Krishgen biosystem, Mumbai, India) and IL-1 Β (KLR0119; Krishgen biosystem, Mumbai, India) were used. Mitochondrial ELISA kits used included ETC complex I [E-BC-K149-M, Elabscience, Maharashtra, India), ETC complex II (E-BC-K150-M, Elabscience, Maharashtra, India), ETC complex III (E-BC-K151-M, Elabscience, Maharashtra, India), ETC complex IV (E-BC-K152-M, Elabscience, Maharashtra, India), ETC complex V [E-BC-K153-M, Elabscience, Maharashtra, India) [26].

### 4.7. Gross Pathological Analysis

After completing the experimental procedures, the animals were humanely euthanized through the administration of sodium phenobarbital. After euthanasia, brains were extracted and preserved in ice-cold saline to maintain tissue integrity. Then, gross pathological evaluations were performed, employing a digital camera to capture detailed visual assessments of both the entire brain and its coronal slices. The analysis focused on the grey matter surrounding these coronal slices, which served as a reference for quantifying the demyelinated volume of each brain segment in cubic millimeters. To evaluate white matter degeneration, the demyelination volume for each coronal segment was calculated using the formula: volume = length × breadth × height. This quantitative approach provided insights into the extent of neuropathological alterations present in the brain tissue [40].

### 4.8. Histopathological Evaluation

For histopathological examination, brains were extracted to isolate key regions including the cerebral cortex, striatum, and hippocampus. These tissues were purified and sliced into 5 mm sections, which were then fixed in 4% paraformaldehyde in PBS (pH 7.4) for 8 to 12 h at room temperature. After fixation, the samples were rinsed with PBS, dehydrated in 70% ethanol, and incubated at 37 °C before embedding in paraffin. The paraffin blocks were sectioned into 10 μm thick slices using a rotary microtome, stained with hematoxylin and eosin (H&E), and analyzed under a fluorescence microscope at 100× magnification to assess neuronal distribution in the cerebral cortex. Additionally, Luxol Fast Blue (LFB) staining was performed to enhance visualization of myelinated regions. The paraffin-embedded sections underwent a multi-step process that included deparaffinization with xylene, hydration with ethanol, and staining overnight with a 0.1% LFB solution at 50 °C. After differentiation with lithium carbonate and dehydration through graded ethanol solutions, the slides were finally examined microscopically to confirm the transformation of gray matter into white matter [41].

### 4.9. Statistical Analysis

The recorded data from various experiments were statistically analyzed using GraphPad Prism software (version 8.0.1, San Diego, CA, USA). A one-way ANOVA with repeated measures, complemented by Tukey’s post-hoc test, was employed to evaluate neurochemical parameters, ensuring a comprehensive comparison across groups. Additionally, a two-way ANOVA followed by Bonferroni’s post-hoc test was utilized to assess differences in neurobehavioral evaluations among treatment groups, allowing for a nuanced understanding of the interactions between factors. The results were graphically represented as means with standard deviations (SD). A significance threshold of *p* < 0.01 was used.

## 5. Conclusions

In conclusion, this study provides strong evidence for the neuroprotective effects of AZI in a rat model of TBI. AZI administration significantly improved behavioral outcomes and attenuated neuronal loss following TBI. These findings suggest that AZI may represent a promising therapeutic option for TBI. However, further research is needed to fully validate these findings in human clinical trials and to elucidate the underlying mechanisms of action.

## Data Availability

Data will be available upon request. The data generated and analyzed during this study are not publicly available due to institutional policies that require restricted access to experimental datasets. Additionally, the data may contain unpublished findings that are part of ongoing research efforts.
